# True Grit: Passion and persistence make an innovative course design work

**DOI:** 10.1371/journal.pbio.3000359

**Published:** 2019-07-18

**Authors:** Anne M. Casper, Sarah L. Eddy, Scott Freeman

**Affiliations:** 1 Department of Biology, Eastern Michigan University, Ypsilanti, Michigan, United States of America; 2 Department of Biological Sciences, Florida International University, Miami, Florida, United States of America; 3 Department of Biology, University of Washington, Seattle, Washington, United States of America

## Abstract

Our first two experiments on adapting a high-structure course model to an essentially open-enrollment university produced negative or null results. Our third experiment, however, proved more successful: performance improved for all students, and a large achievement gap that impacted underrepresented minority students under traditional lecturing closed. Although the successful design included preclass preparation videos, intensive active learning in class, and weekly practice exams, student self-report data indicated that total study time decreased. Faculty who have the grit to experiment and persevere in making evidence-driven changes to their teaching can reduce the inequalities induced by economic and educational disadvantage.

## Abstract 2

This Community Page article describes evidence-based changes to the design of a large-enrolment introductory biology course at a regional comprehensive university; exam scores increased and failure rates decreased for all students, with under-represented minority students—predominantly African-Americans—benefiting disproportionately.

A recent meta-analysis found that students in lecture-based science, technology, engineering, and mathematics (STEM) courses are 1.5 times more likely to fail, on average, than students in courses with at least some active learning [1]. Furthermore, some course designs with active learning reduce or eliminate achievement gaps that have traditionally harmed underrepresented or underprepared students [2,3]. In courses with active learning, students are not just listening during class but, rather, engaged in doing meaningful learning activities. Based on results like these, calls for the use of active learning in STEM classes have reached a crescendo [4].

Individual results vary, however: some implementations of active learning produce no change or even reduced student performance compared with lecturing [1]. How can faculty make sure they don’t try active learning and fail?

Our answer to that question is straightforward. To implement evidence-based teaching strategies effectively, faculty will need to pursue the same approach that they use when adopting a new technology in their bench or field research. Specifically, instructors need to follow the literature closely, get initial training from experienced practitioners, start with limited trials, be prepared for disappointing results at first, engage with colleagues who can provide feedback and advice, and be willing to make changes (via repeated attempts over time) that adapt the technique to their study system—in this case, to their course material and student population [5,6].

## What is a high-structure course?

Impressed with accumulating evidence on the efficacy of active learning, the first author set out to implement a high-structure course design with the goal of improving student outcomes compared with her lecture-based class. A high-structure design requires students to:

- learn basic content on their own prior to class, with a quiz based on assigned readings or videos;
- apply the content to higher-order problems through active-learning exercises during class, usually by working with peers and getting feedback from the instructor [1]; and
- test their understanding after class by taking a low-stakes quiz or practice exam.

In essence, a high-structure course brackets intensive active learning during face-to-face interactions in the classroom with individual preparation prior to class and self-testing after [3]. Flipped models are similar, but they leave out the self-testing element that occurs after class and prior to high-stakes exams.

Designs like this have been extremely effective in the hands of instructional faculty who work at flagship public universities [2,3]. But can they be successful when implemented by faculty who are hired and promoted primarily based on their productivity in bench- or field-based research versus educational research? And can they work at regional, comprehensive (master’s-granting) universities—for which admission requirements are minimal and students are often underprepared?

We set out to answer these questions at Eastern Michigan University (EMU), a regional, master’s-granting public university that admits almost all applicants to its undergraduate program. Nearly half of EMU students are from low-income backgrounds, 35% transferred in from other institutions, and 35% are underrepresented minorities (S1 Text). The course involved was BIO 110, which is the first in a two-semester introductory biology sequence for undergraduate science majors. BIO 110 is offered every semester, meets for two 75-minute class sessions each week, includes a weekly 4-hour lab, and has a pre- or corequisite of college algebra. Course content includes biological molecules, cell structure and function, energetics, cell division, mendelian and population genetics, evolution, and ecology.

## Gathering evidence: Comparing apples with apples

The first author partnered with the other authors on this study because she was committed not only to changing how BIO 110 was taught but also to collecting and analyzing data on student outcomes compared with the traditional mode of instruction. The goal was to change one or more aspects of course design, evaluate the impact on student performance, and make further adjustments if results were disappointing or iterate if results were inconclusive or promising.

To study whether course innovations impact student performance, our team focused on controlling for the instructor, the students, and the test.

- As we tried different iterations of the high-structure course design, we kept constant the instructor, total student contact time in lecture sessions and labs, and course content. The first author taught the course in a traditional fashion five times before implementing aspects of high structure for the first time, and we used the last two of these terms as the lecture comparison group (the control group; Table 1). As a result, it is unlikely that differences in student outcomes over time resulted from the instructor simply gaining experience with the material.
- To control for possible among-term variation in the academic ability or preparation of EMU students, we collected college admission test (ACT) scores and distilled them into a composite index. We then used this index in statistical analyses of course performance (S2 Text). This made us confident that changes in student outcomes were not the result of changes in student ability or preparation.
- We controlled for differences in exam difficulty in two ways: (1) we used Bloom’s taxonomy of learning to characterize the cognitive challenge of each term’s exams, and (2) we calculated the fraction of exam questions that were reused from exams that were not returned to students and thus were identical across terms. Both analyses showed that there was no change in exam difficulty over the course of the study (S2 Text). This result gave us confidence that increases in student performance weren’t due to assessments getting easier over time.

**Table 1:** Changes in course design.

		During class			
Experimental group	Before class	Overall style	Clickers	In-class questions posed by instructor	After class
Control (two semesters, four total class sections; *N* for each section = 66, 143, 56, 144)	Recommended textbook reading	Lecture	For points; answered in groups	Few, answered by volunteers	Online homework, 15–30 questions, one per week
Experiment 1 (one semester, two total class sections; *N* for each section = 60, 150)	No change	No change	No change	No change	Online practice exam with time limit, 15 questions, one per week
Experiment 2 (one semester, two total class sections; *N* for each section = 59, 154)	Required textbook reading, online reading quiz, short-answer homework due at beginning of class (hand-graded by GAs)	Lecture-free; group worksheets and activities	For points; answered alone first, then peer instruction	Many, random call for answers	Online practice exam without time limit, 15 questions, one per week
Experiment 3 (three semesters, four total class sections; *N* for each section = 55, 88, 102, 153)	Required video, closed-notes short-answer quiz over video at beginning of class (peer-graded)	No change	No change	No change	Online practice exam without time limit, 20–30 questions, one per week

In each case, “No change” indicates no changes from the previous version of the class.

Abbreviation: GA, graduate student assistant

Our data also indicated that the exams were not easy. The analysis based on Bloom’s taxonomy showed that exam questions in the study terms averaged at slightly above the conceptual level, which is higher than the average reported for introductory biology exams in the largest national sample in the literature [7].

To measure student outcomes, we evaluated both exam scores and failure rates—the latter calculated as the percentage of students who withdrew or received a D or F grade (“DFW rate”). Because high-structure courses add a large number of course points based on participation, we were concerned about grade inflation—specifically, the hypothesis that the DFW rate could go down in a high-structure course simply because struggling students amass participation-based nonexam points. To test this hypothesis, we analyzed simple linear regressions of the percent of exam points earned on total points earned and, thus, grade. The results suggest that grades were not inflating because of nonexam points. In fact, compared with terms with traditional instruction, exams seemed to be more important in explaining variation in final grade as we implemented additional aspects of high structure (S2 Text).

## If at first you don’t succeed …

During experiment 1, the first author taught BIO 110 by adding one element of high structure to the course. Specifically, she replaced the traditional weekly homework assignment, consisting of about 30 questions in various formats, with an autograded, online, weekly practice exam. Practice exams differed from the homework assignments given in previous semesters in three ways: (1) they consisted of 15 multiple-choice questions drawn from old exams, (2) they could only be opened once instead of multiple times, and (3) all questions had to be completed within 30 minutes instead of having no time limit. During class, the first author frequently encouraged students to study the week’s material prior to taking each practice exam just as they would study for a regular exam. Our rationale for implementing the practice exams was that the incentive to “keep up” with a bit of exam-style studying every week throughout the semester, coupled with regular exposure to actual exam questions, would improve student success on regular exams. All other aspects of the course during this experiment 1 were the same as the control semesters. The experiment was motivated by the hypothesis that practice exams should have the highest benefit for students with the lowest cost of implementation, as they align closely with actual exams and are administered online.

The result? Both measures of student performance went down: exam scores decreased and DFW rates increased. Clearly, substituting a timed practice exam for a packet of study questions did not benefit students. Adding just one of three elements of high structure was not helpful. At EMU, the lowest-cost element of high structure, in terms of instructor effort, had a high cost to students.

## Try …

In response, the first author committed to implementing all three aspects of a high-structure course to replicate the previously published designs as closely as possible. Prior to the start of experiment 2, she used funds from an internal EMU award for an in-person visit to the University of Washington to learn through observing the classroom dynamics of her collaborators. During the experiment 2 semester, students in the first author’s course prepared for each class session by (1) completing an assigned reading from the textbook, (2) taking an online, autograded quiz over the prereading, and (3) answering a set of open-response, guided-reading questions modeled on materials developed during a successful implementation of high structure [2]. Students turned in their written answers to the guided-reading questions at the beginning of each class session; graduate teaching assistants graded these responses during class, entered grades, and returned the assignments at the end of class.

During class, the instructor’s goal was to minimize the time spent on lecturing. Instead, students (1) worked in groups to complete activities and worksheets; (2) answered 3–5 graded clicker questions in the evidence-based, peer-instruction format [8]; and (3) were called on, at random, to respond to frequent verbal questions posed by the instructor. In addition, students completed autograded practice exams identical to those used in experiment 1, except that the time limit was removed. Students could also open their practice exams multiple times, instead of having to complete it in one sitting.

The result? There was no change in student exam performance or DFW rates relative to the control (lecture) terms. The high-structure format—modeled closely after course designs that had been successful at flagship public universities [2,3] and at a highly selective private institution [9]—was not helping EMU students.

## Try again

Experiment 2 provided a key observation: the first author repeatedly found students were unable to carry out the planned in-class activities. This observation resonated with prior studies showing that elaborative interrogation, which occurred during experiment 2’s in-class activities, only benefits students with strong background knowledge [10]. BIO 110 students appeared to be struggling with the critically important preclass preparation phase of the high-structure model. We hypothesized that this problem was due to a deficit in reading comprehension caused by economic and educational disadvantage. A comparison of average reading comprehension scores on college admission tests supported this hypothesis: at one of the flagship universities that pioneered the high-structure model, average scores for incoming freshmen were above the 80th percentile, whereas EMU students, in contrast, averaged in the 50th percentile (S1 Text).

The first author responded by teaching BIO 110 using a modified version of the experiment 2 format. The major change was that before every class meeting, students watched a 15-minute video lecture created by the instructor and then reviewed 15–20 questions on the material. The videos were meant to explain the most important vocabulary and concepts relevant to each face-to-face meeting; each took about 4 hours to produce. The first author used teaching release time she received through an internal EMU award to create the video lectures. Each class session then began with a closed-notes, short-answer quiz with two questions from the 15–20 provided. Quizzes were peer-graded immediately and returned to their owner. Each student then entered their quiz grade using their clicker (personal response system). Quizzes were collected, and the accuracy of student-entered scores was spot-checked after class; the penalty for entering an incorrect quiz score entry was a 15% deduction in points from the overall quiz grade for the semester. Other aspects of the course were identical to experiment 2, except that the number of practice exam questions was expanded to 20–30 each week.

The changes in course design that occurred over the three experiments (Table 1) are backed by data from a classroom observation tool [11] (S3 Text). In experiment 2 and experiment 3, (1) the instructor made extensive use of random call as a method for keeping students accountable for in-class group work [12]; (2) preclass preparation was used to help students become grounded in relevant vocabulary and concepts prior to class [13], and the instructor made a large increase in the cognitive challenge of in-class activities [14]; (3) clicker use became more evidence-based with the implementation of peer instruction [8]; and (4) students spent most of each class period discussing problems with peers. An example of a typical class session during experiment 3 is provided (S6 Text, S1 Video, S1 PowerPoint).

**Fig 1:**
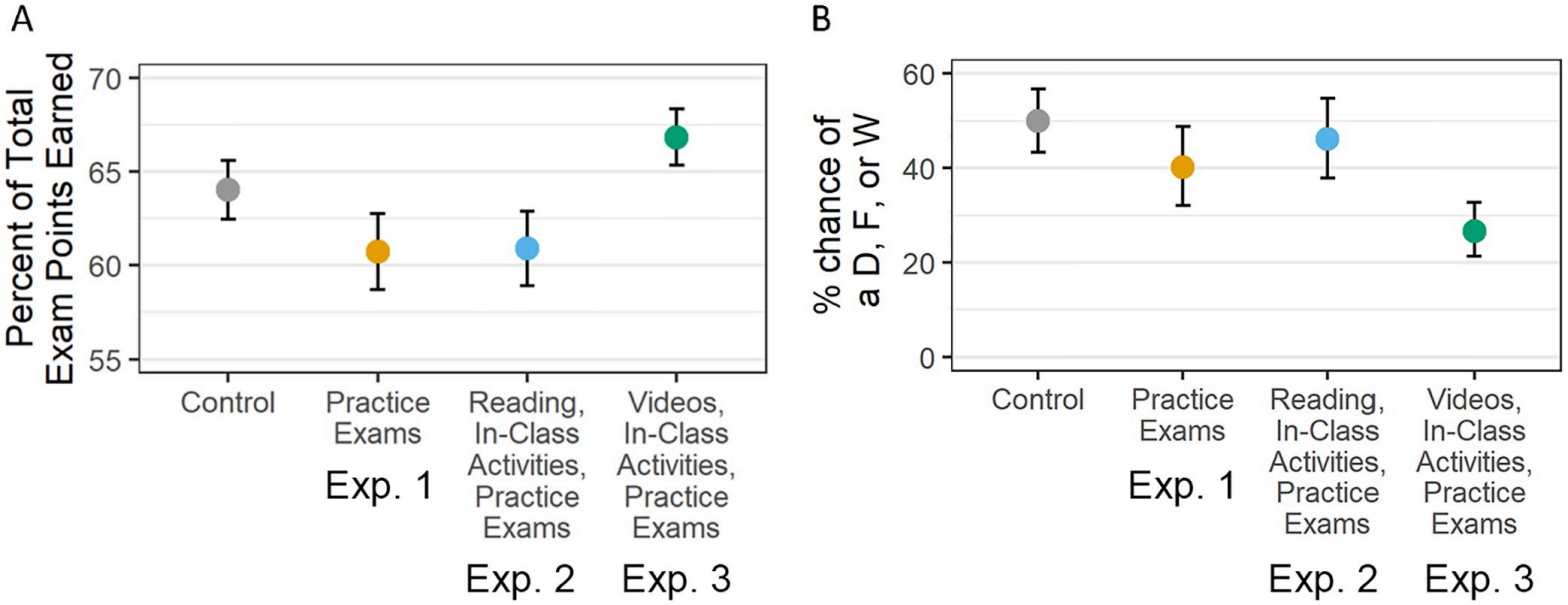
Students in Exp. 3 had increased exam performance and lower failure rates. The data points are model-predicted average exam points earned on each exam (A) and percent chance of earning a D or F or withdrawing from the course (B) (S1 Data). Predictions are for students with average ACT scores in the study sample. Error bars represent the standard error around the estimate. Exp., experiment; W, withdrawal.

The result? Controlling for ACT scores, gender, and transfer status, exam scores during experiment 3 increased by an average of 3.8% (Fig 1A; S4 Text; S1 Data). Controlling for underrepresented minority (URM) status, DFW rates saw a highly significant drop (Fig 1B; S4 Text; S1 Data); raw DFW rates went from 48% to 25% (S4 Text; S1 Data). Even though exams were equivalent in difficulty, students in experiment 3 did much better on average than students in the control, lecture-based semesters—a result consistent with gains documented in a recent meta-analysis of active-learning interventions [1].

**Fig 2:**
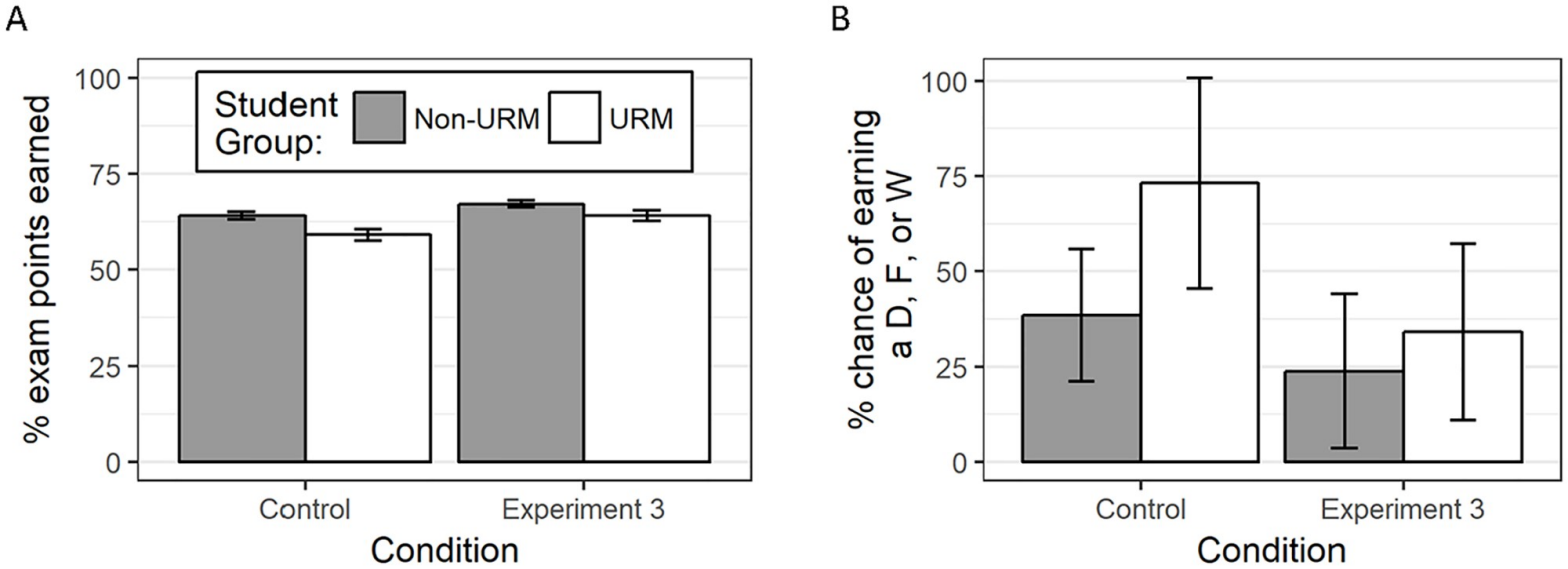
Achievement gaps shrank in experiment 3. The bars are model-predicted average exam points earned on each exam (A) and percent chance of earning a D or F or withdrawing from the course (B) (S1 Data). Predictions are for students in these courses, with the average score for the summary variable based on ACT reading, math, English, and science reasoning scores. Error bars represent the standard error around the estimate. URM, underrepresented minority; W, withdrawal.

But just as—or even more—important, our data showed that the course design in experiment 3 had a disproportionately beneficial impact on URM students in BIO 110—over 85% of whom self-identify as African American. In experiment 3, persistent achievement gaps closed (Fig 2; S1 Data). Under lecturing, URM students with average ACT scores had a 73% chance of failing; in experiment 3, this fell to 34%. In the regression model of failure rates, the interaction between experiment 3 as a treatment and URM status was statistically significant (S4 Text; S1 Data). Although this term was not significant in the regression model of exam scores when controlling for ACT (S4 Text; S1 Data), it was retained in the best model—providing strong support for an interaction between treatment and URM status in explaining exam score performance. In addition, the experiment 3 by URM status interaction was statistically significant in the model without an ACT term, which included 187 students with missing ACT data. That analysis provides a much larger sample size and conforms to the “transcriptable” performance that students see, and it indicates that all students did 3.9% better on exams in experiment 3 compared with terms with lecturing, with URM students getting an additional 5.2% bump (S4 Text; S1 Data). Taken together, the exam score analyses suggest that the dramatic impact on failure rates for URM students was driven by disproportionately better performance on exams. Why do URM students benefit more than other subpopulations? Active learning increases social integration, particularly for underrepresented groups [2], and a sense of belonging is important for success in the sciences. However, the in-class active learning was the same in our experiments 2 and 3, yet we only observed gains in student success in experiment 3. This suggests that the format of preclass information delivery—video lectures in experiment 3—is particularly important for the population of students at EMU. In effect, the experiment 3 course design helped underrepresented students overcome deficits in preparation caused by the circumstances of their birth. This study succeeded in replicating reductions in achievement gaps that have occurred at selective and highly selective institutions [2,3,9].

## True grit

In this study, we describe hypothesis-driven adjustments to the course design, intense effort, and several iterations to translate an evidence-based course developed at flagship, research-intensive universities to the context of an open-enrollment, regional public university, at which low-income students are a near majority. Our initial and repeated failure will be familiar to any scientist: it is analogous to the process that researchers go through when adopting a new technique in bench or field work. It is almost unheard-of for a new protocol, reagent, or piece of equipment to work the first time. Research groups routinely have to make adjustments to fit their apparatus, goals, and study system.

In this case, the key adjustment was changing from preclass reading assignments and online prep quizzes to preclass video lectures and in-class prep quizzes. This change was motivated by two key factors: (1) the observation that open-enrollment institutions host a higher percentage of underprepared students than selective institutions and (2) the hypothesis that, even if underprepared students have difficulty extracting information from textbook reading at this early stage in their careers, they can prepare for class effectively by viewing minilectures that are authored by the instructor of record and that focus on key vocabulary and concepts.

**Fig 3:**
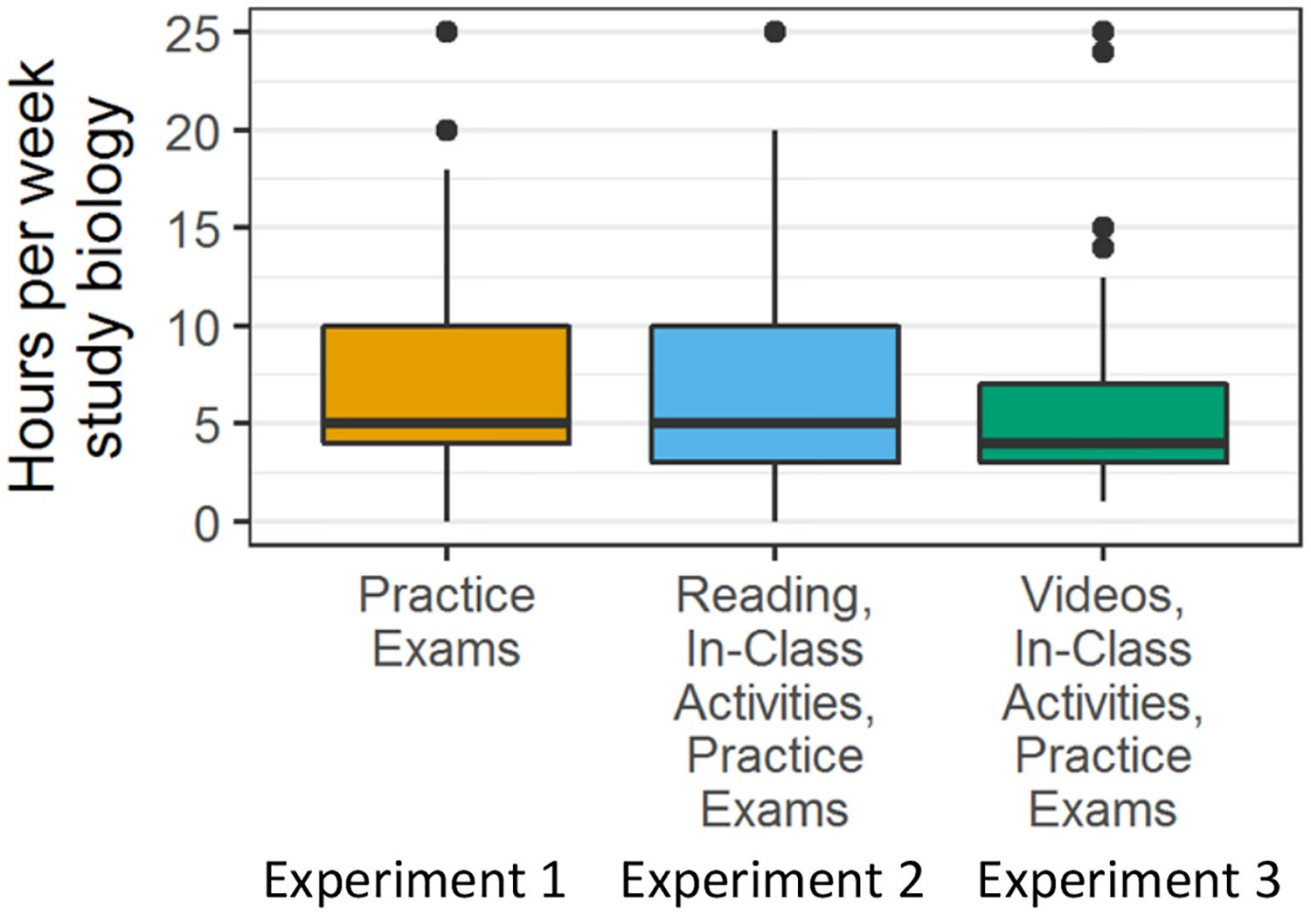
Students in experiment 3 spent less time studying. The boxes indicate the first quartile, median, and third quartile of the raw data (not model-predicted) of self-reported time spent studying per week for introductory biology; the whiskers represent 1.5 times the interquartile range; the dots are data points outside this range (S1 Data). Although we do not have data from control semesters, the course format was identical to that of experiment 1 except for replacing homework problems with a timed practice exam (Table 1).

Our data yielded two additional insights. First, the elements of course structure that were added in experiments 2 and 3 did not burden students in terms of time demands. Instead, self-reported time spent studying outside of class decreased—even though student performance increased (Fig 3; S5 Text; S1 Data). This result suggests that the additional structure helped students become much more efficient in their studying. Second, student evaluations of teaching improved overall. As Table 2 shows, the percentage of students rating the course and/or the instructor as above average increased in most cases, implying that the students recognized the value of the high-structure course design and appreciated the instructor’s efforts to improve their performance.

**Table 2:** Student evaluations of teaching.

**Overall rating of course**			
Semester	Percent of students selecting much below or below average	Percent of students selecting average	Percent of students selecting above or much above average
Control semester 1	11	25	64
Control semester 2	13	27	60
Experiment 1	9	23	68
Experiment 2	14	30	55
Experiment 3 semester 1	8	19	73
Experiment 3 semester 2	2	13	85
Experiment 3 semester 3	5	36	58
**Overall rating of teaching effectiveness**			
Semester	Percent of students selecting much below or below average	Percent of students selecting average	Percent of students selecting above or much above average
Control semester 1	10	26	64
Control semester 2	10	18	72
Experiment 1	13	15	73
Experiment 2	13	19	68
Experiment 3 semester 1	4	13	82
Experiment 3 semester 2	2	13	85
Experiment 3 semester 3	12	8	79

Course evaluation response rates were 58% in control term 1, 55% in control term 2, 61% in the experiment 1 term, 68% in the experiment 2 term, 65% in experiment 3 term 1, 71% in experiment 3 term 2, and 75% in experiment 3 term 3.

It is important to recognize, though, that adopting evidence-based teaching practices is challenging for STEM faculty who identify primarily as bench- or field-based researchers [15]. Changing a traditional course to a high-structure model with good fidelity of implementation requires time, institutional support, intentional effort to gain expertise and mentoring, and perseverance. The first author particularly needed time and institutional support in the semesters preceding her major course structure changes, to facilitate in-person learning from experienced instructors, and to create instructional videos. After this initial investment, however, the high-structure course has not been more time-consuming for the first instructor to run than her traditional lecture course. The trait of perseverance when making these changes may be underappreciated, as surveys have shown that one-third of faculty who try evidence-based teaching strategies give up after an initial attempt [5]. Adapting techniques and retrying after initial failures requires a deep personal commitment, as well as emotional support from skilled peers. Using this project as a model, we hope other faculty will make class-based research an important part of their overall research and teaching portfolio—making observations about their students, posing testable hypotheses, performing experimental interventions, and collecting data on outcomes.

This project succeeded in improving exam scores, lowering failures rates for all students, and reducing an achievement gap affecting URM students. These outcomes were immensely rewarding but only possible because of professional grit [16]. Perseverance in the classroom is fueled by a passionate commitment to student success, just as perseverance at the lab bench is fueled by a passionate desire to know.

## Methods

### Ethics statement

This research was conducted after review and approval by the University of Washington Human Subjects Division, application 38945, and the EMU Human Subjects Review Committee, protocols numbered 867551–1, 867551–2, and 131005.

## Supporting information

S1 Text Institutional context and student demographics. (DOCX)

S2 Text Controlling for variation in student ability and exam difficulty. (DOCX)

S3 Text Analyzing changes in course structure over time. (DOCX)

S4 Text Analyzing changes in student performance. (DOCX)

S5 Text Time-on-task survey and student evaluations of teaching. (DOCX)

S6 Text Example of a typical class session during experiment 3. (DOCX)

S1 Video Preclass video that accompanies the class session described in S6 Text. (MP4)

S1 PowerPoint PowerPoint that accompanies the class session described in S6 Text. (PDF)

S1 Data Individual quantitative observations that underlie the data summarized in the figures and results. (XLSX)

## Abbreviations

DFW rate: percentage of students who withdrew or received a D or F grade
EMU: Eastern Michigan University
GA: graduate student assistant
STEM: science, technology, engineering, and mathematics
URM: underrepresented minority
W: withdrawal
