# Supplementary material for: True Grit: Passion and persistence make an innovative course design work
Source: PLoS Biol. 2019 Jul 18;17(7):e3000359. doi: 10.1371/journal.pbio.3000359 (PMC6667208; doi:10.1371/journal.pbio.3000359)
Supplement: S1 Text — (DOCX) [file pbio.3000359.s001.docx]

**S1 Text. Institutional context and student demographics.**

We tested the hypothesis that a high-structure pedagogical approach that was initially developed, implemented, and tested in introductory biology courses at the University of Washington [1] could be transferred to the equivalent course at Eastern Michigan University, with similar beneficial outcomes for all students and especially for underrepresented students. The University of Washington is a flagship public research-intensive university that admits about 53% of applicants to its undergraduate program. Eastern Michigan University is a regional master’s-granting public university that admits almost all applicants to its undergraduate program.

Undergraduates at the two institutions differ in key characteristics (see Table S1). Perhaps the most important observation is that for a standard college admission test—the ACT—the interquartile range of scores from the two student populations do not overlap, with Eastern Michigan’s being lower. In addition, Eastern Michigan has twice as many students from low-income backgrounds (48% versus 24%), 75% more students who transfer into the university instead of being admitted as freshmen, and almost triple the percentage of under-represented minorities (35% versus 13%).

**Table S1. Undergraduate Profile Comparison.**

American College Testing (ACT) is a college-readiness exam used as an admission criterion. Under-represented minorities (URMs) are African-American, Latino/a, Pacific Islander/Native Hawaiian, or Native American. Pell grants are U.S. government scholarships available to students from low-income backgrounds. “Transferred in” refers to students who transferred into the bachelor’s-granting institutions studied here from other institutions—usually community colleges.

|  | Eastern Michigan University | University of Washington-Seattle |
| --- | --- | --- |
| ACT interquartile range | 19 – 25 | 25 – 31 |
| Underrepresented minority (URM) students | 35%^a^ | 13%^b^ |
| Pell grant eligible | 48% | 24% |
| Transferred in | 35% | 20% |
| First-year students in campus dorms or fraternities/sororities | 23% | 72% |
| Enrolled full-time | 66% | 90% |
| Graduate degrees offered in Biology | Master’s | PhD |

^a^ 54% of URM students are Black/African American

^b^ 55% of URM students are Latino/a

[1] Haak DC, HilleRisLambers J, Pitre E, Freeman S (2011). Increased course structure and active learning reduce the achievement gap in introductory biology. *Science* *332*(6034), 1213-1216. DOI: 10.1126/science.1204820.
