# Supplementary material for: True Grit: Passion and persistence make an innovative course design work
Source: PLoS Biol. 2019 Jul 18;17(7):e3000359. doi: 10.1371/journal.pbio.3000359 (PMC6667208; doi:10.1371/journal.pbio.3000359)
Supplement: S2 Text — (DOCX) [file pbio.3000359.s002.docx]

**S2 Text. Controlling for variation in student ability and exam difficulty.**

We used data from the EMU Office of Institutional Research and Information Management to document the ACT scores that each student submitted in their application to the university (S9 Data). Preliminary analyses indicated correlations ranging from 0.54 to 0.70 among ACT English, math, reading, and scientific reasoning sub-scores, so we performed a principal components analysis. The PCA recovered a first factor, comprised of roughly equivalent loadings from all four sub-scores, that explained 72% of the total variance in ACT scores. We computed this PC1 value, which we called ACT-composite, for each student and used this value as a covariate in regression analyses.

To test the hypothesis that the DFW rate could go down in a high-structure course simply because struggling students amass participation-based non-exam points, we performed simple linear regressions of exam points on final grade. Regressions report an *R^2^* value, which indicates the percentage of variation explained by the predictor variable(s). In this analysis, our *R^2^* value summarizes the percent of variation in final grade that is explained by exam performance. We found that the *R^2^*s were 0.60 and 0.64 for the two control terms, 0.68 in the initial implementation of high-structure (Experiment 1), 0.69 for the second type of high structure (Experiment 2), and 0.81, 0.69, and 0.77 for the three implementations of the final approach to high structure (Experiment 3).

To test the hypothesis that student outcomes changed as a result of changes in exam rigor over time, four experienced researchers—none of whom was a course instructor—worked in teams of two to independently assign a Bloom’s taxonomy level 1-6 to 33% of all exam questions used during each semester in the study [1]. Each semester, there were four exams containing 40 questions each, for a total of 160 exam questions per semester. There were seven semesters in the study; two control semesters, one Experiment 1 semester, one Experiment 2 semester, and three Experiment 3 semesters. For each semester in the study, 33% of all exam questions were Bloomed – that is, 53 exam questions from each semester were Bloomed. The questions that were Bloomed were chosen at random and the raters were blind to the semester each question was posed. The rater-teams then met as a pair to discuss the scores and reach consensus. From these data and the points per question, we computed a weighted Bloom’s index summarizing the average cognitive challenge of exams in each semester [2]. A weighted Bloom’s index of 33.3 represents an exam consisting entirely of questions at the Conceptual Level; an index of 50.0 indicates an exam consisting entirely of questions at the Application level [3].

As Table S2 shows, the exams in this study all averaged at above the conceptual level, which exceeds the average documented in the largest national sample to date [4]. An ANOVA showed that there was no difference in average Bloom’s level over the seven terms in the study (*F* = 0.46, df 6, *p* = 0.84).

Because the instructor did not return any exams and subsequently was able to re-use some exam questions, we also calculated the fraction of exam questions that were identical in each of the experimental terms to at least one of the control terms. As the data in Table S2 indicate, most of the exam questions used over the course of the study were identical. The percentage of exam questions that were the same as questions used during the control terms ranged from 65-100%.

**Table S2. Exam characteristics.**

| **Treatment** | **Percent of questions identical to control** | **Weighted Bloom’s Index** |
| --- | --- | --- |
| Control | --- | 37.7 |
| Experiment 1 | 100 | 36.2 |
| Experiment 2 | 68 | 37.1 |
| Experiment 3 | 65 | 35.0 |

**References**

[1] Crowe, A., Dirks, C., & Wenderoth, M. P. (2008). Biology in bloom: Implementing Bloom’s taxonomy to enhance student learning in biology. *CBE—Life Sciences Education* *7*(3), 368-381. DOI: 10.1187/cbe.08–05–0024.

[2] Freeman, S., O'Connor, E., Parks, J. W., Cunningham, M., Hurley, D., Haak, D., Dirks, C., & Wenderoth, M. P. (2007). Prescribed active learning increases performance in introductory biology. *CBE—Life Sciences Education* *6*(2), 132-139.

[3] Freeman, S., Haak, D., & Wenderoth, M. P. (2011). Increased course structure improves performance in introductory biology. *CBE—Life Sciences Education* *10*(2), 175-186. DOI: 10.1187/cbe.10-08-0105.

[4] Momsen JL, Long TM, Wyse SA, Ebert-May D (2010). Just the facts? Introductory undergraduate biology courses focus on low-level cognitive skills. *CBE—Life Sci Ed* *9*(4), 435-440. DOI: 10.1187/cbe.10-01-0001.
