# Supplementary material for: True Grit: Passion and persistence make an innovative course design work
Source: PLoS Biol. 2019 Jul 18;17(7):e3000359. doi: 10.1371/journal.pbio.3000359 (PMC6667208; doi:10.1371/journal.pbio.3000359)
Supplement: S3 Text — (DOCX) [file pbio.3000359.s003.docx]

**S3 Text. Analyzing changes in course structure over time.**

To test the hypothesis that class sessions changed as intended between the Control condition and Experiments 2 and 3, we selected three video-plus-audio recordings chosen at random from class sessions conducted during one of the two Experiment 1 semesters, which were identical to the Control terms, and three recordings chosen at random from class sessions during the Experiment 2 semester, which were identical to the Experiment 3 terms. We analyzed each recording using the Practical Observation Rubric To Assess Active Learning, or PORTAAL, classroom observation tool [1]. The PORTAAL classroom observation tool quantifies the use of best practices for implementing active learning in the following dimensions, 1) practice, 2) logic development, 3) accountability, and 4) apprehension reduction. The first dimension, practice, is quantified through the amount and quality of activities in which students are working through content, such as through clicker questions or group work. The dimension of logic development is quantified through the percent of activities that promote higher-order thinking skills. Accountability is quantified through the amount of teacher-provided incentives that motivate students to participate in activities during class, such as by awarding points for activities or by using random call for in-class questions. Apprehension reduction is quantified through the strategies used by instructors to reduce students’ fear of participation, such as by giving praise or encouragement after students answer in-class random call questions. The values shown in Table S3 were selected because they reflect the changes in course design summarized in Table 1. The shifts in the scores indicate increased use of evidence-based, student-centered practices in the experimental terms versus the control terms.

Most of the changes documented in Table S3 were due to the instructor adopting two key aspects of evidence-based active learning: the peer instruction framework for clicker questions [2,3,4] and random call to solicit student ideas after group work on a problem [5]. In this context, it is interesting to note that the instructor had incorporated non-evidenced based approaches to clicker use and classroom questions during the control terms. During these semesters, the instructor asked a total of 3-5 clicker questions at intervals during each class. Students were allowed to talk with each other while the question was open for response, and course points were awarded for correct responses. The instructor also posed occasional questions verbally and called on student volunteers. Recent research has shown that this non-evidenced-based approach to instituting active learning is extremely common [6]. The data in Table S3 shows a marked difference in student engagement in the non-evidenced-based versus evidence-based implementation of active learning.

**Table S3. Selected changes in classroom practice quantified via PORTAAL**

| **PORTAAL category** | **PORTAAL metric** | **Control** | **Experiment 2** |
| --- | --- | --- | --- |
| Accountability | Percent of activities with random call | 4% | 68% |
| Logic development | Percent of activities with higher-order skills | 38% | 60% |
| Logic development | Percent of activities where students thought alone first | 0% | 48% |
| Logic development | Percent of activities where students discuss answers with peers | 29% | 63% |
| Student practice | Percent of class time with students talking | 26% | 52% |

**References**

[1] Eddy SL, Converse M, Wenderoth MP (2015). PORTAAL: a classroom observation tool assessing evidence-based teaching practices for active learning in large science, technology, engineering, and mathematics classes. *CBE—Life Sciences Education* *14*(2), 1-16. DOI: 10.1187/cbe.*14*-06-0095.

[2] Mazur, E. (1997). Peer Instruction. *Upper Saddle River NJ: Prentice Hall.*

[3] Crouch CH, Mazur E (2001). Peer Instruction: Ten years of experience and results. *American Journal of Physics 69*(9), 970-977. DOI: 10.1119/1.1374249.

[4] Vickrey T, Rosploch K, Rahmanian R, Pilarz M, Stains M (2015) Research-based implementation of peer-instruction: a literature review. *CBE-Life Sci Ed* 14(2):1-11; DOI:10.1187/cbe.14-11-0198.

[5] Eddy, S. L., Brownell, S. E., & Wenderoth M. P. (2014). Gender gaps in achievement and participation in multiple introductory biology classrooms. *CBE—Life Sciences Education* *13*(3), 478-492. DOI: [10.1187/cbe.13-10-0204](https://dx.doi.org/10.1187%2Fcbe.13-10-0204).

[6] Stains M, Harshman J, Barker MK, Chasteen SV, Cole R, DeChenne-Peters SE, Eagan MK, Esson JM, Knight JK, Laski FA, Levis-Fitzgerald M, Lee CJ, Lo SM, McDonnell LM, McKay TA, Michelotti N, Musgrove A, Palmer MS, Plank KM, Rodela TM, Sanders ER, Schimpf NG, Schulte PM, Smith MK, Stetzer M, Van Valkenburgh B, Vinson E, Weir LK, Wendel PJ, Wheeler LB, Young AM (2018) Anatomy of STEM teaching in North American universities. Science 359(6383): 1468-1470, DOI: 10.1126/science.aap8892.
