# Supplementary material for: True Grit: Passion and persistence make an innovative course design work
Source: PLoS Biol. 2019 Jul 18;17(7):e3000359. doi: 10.1371/journal.pbio.3000359 (PMC6667208; doi:10.1371/journal.pbio.3000359)
Supplement: S4 Text — (DOCX) [file pbio.3000359.s004.docx]

**S4 Text. Analyzing changes in student performance.**

We collected course performance data from the instructor’s gradebook and demographic and academic history data from the University’s Office of Institutional Research and Information Management (S9 Data). Prior to analysis, we removed students who did not consent to participate in the study, take at least three exams, or report ACT scores to the University. A priori, we also removed a small number of atypical individuals who enrolled as guest, post-baccalaureate, or high school students, along with a small number of students who self-identified as mixed race or who did not report ethnicity. Pacific Islander, Native American, Latino/a, and African-American students were grouped in the category underrepresented (URM); students of other self-declared ethnicities (White and Asian-American) were considered non-URM. Thirty-four percent of the students in the study were URM, with 85% of URM students self-identifying as African-American. A summary of raw ACT scores and exam performance for the Control and Experimental treatments, not controlled for variation in student characteristics, is provided in Table A.

**Table A. Raw ACT scores and exam performance by treatment**

| **Treatment** | **Class sections** | **Median composite ACT score (first and third quartiles)** | **Median percent of total exam points (first and third quartiles)** |
| --- | --- | --- | --- |
| Control | 2 semesters, 4 total class sections; N for each section = 66, 143, 56, 144 | **21 (19—24)** | **64 (50—77)** |
| Experiment 1 | 1 semester, two total class sections; N for each section = 60, 150 | **21.5 (19—24)** | **60 (48—72)** |
| Experiment 2 | 1 semester, two total class sections; N for each section = 59, 154 | **21 (19—24)** | **62 (51—73)** |
| Experiment 3 | 3 semesters, four total class sections; N for each section = 55, 88, 102, 153 | **22 (20—25)** | **69 (58—78)** |

To evaluate changes in student success, we first analyzed exam scores from the two Control semesters and each of the Experimental treatments. Each exam during the study had 40 2.5-point questions, for a total of 100 points. There were three exams in one of the Control terms; in the other Control term and in all the Experimental terms, there were four exams. When we plotted the percent of total exam points earned for each term in the study, the distributions were Gaussian.

Because total exam points varied among terms, we calculated the percent of exam points earned from the total possible for each student and used this outcome variable in multiple linear regression models. We also used failure rate, quantified as the percentage of students earning a D or F grade or withdrawing sometime after the first exam in each semester, as a response variable in logistic regressions.

*Regression analyses on exam points, for all students*

Data analysis began with an exploratory phase consisting of simple linear regressions with each predictor and response variable. Examining the percentage of overall variation explained suggested that several variables other than treatment influenced exam performance, including gender, URM status, and whether students were “native” to EMU or transferred in from another institution; in a typical semester, about 15% of students were transfers.

To assess the impact of the treatments on exam scores for all student, regardless of URM status, we constructed a full model where we represented the treatment variable as scores from the four conditions—the control and the three experimental interventions. Stated another way, the variable Treatment had three states corresponding to Experiments 1-3, contrasting with the control as the reference condition. We combined the data from the two control semesters and from the three Experiment 3 terms. As noted, the full model also included the variables that explained a significant amount of variation in the simple regressions. Once we had constructed this full model, we performed forward model selection based on AIC values to identify any interactions between the treatment and other predictor variables that influenced student outcomes [1]. None of the interaction terms led to lower AIC values, and in all instances of model equivalence, we chose the model with the fewest predictor variables. The full model was:

*Percentage exam points earned ~ ACT-composite + Gender +*

*Transfer status + Treatment*

In all models, the reference values were Female for Gender, meaning that positive estimates (betas) indicate that males have higher values than females; No for Transfer status, meaning that positive estimates indicate that transfer students have higher values than “native” students; and Control for Treatment, meaning that positive estimates indicate that students in the treatment have higher values than students in the Control terms. Residual plots indicated that the data met the assumptions of normality.

A summary of the regression statistics is provided in Table B.

**Table B. Regression statistics: predicting exam scores across four treatments.**

This is the analysis plotted in Figure 1A.

Estimate Std. Error t value Pr(>|t|)

(Intercept) 23.87 2.63 9.06 < 0.001

ACT composite 0.27 0.02 17.10 < 0.001

Gender 3.01 0.94 3.22 0.001

Transfer 2.71 1.27 2.14 0.03

Experiment 1 -3.30 1.31 -2.51 0.01

Experiment 2 -3.13 1.29 -2.43 0.02

Experiment 3 2.82 1.12 2.53 0.01

Residual standard error: 11.65 on 702 degrees of freedom

Multiple R-squared: 0.43

Adjusted R-squared: 0.43

F-statistic: 75.69 on 7 and 702 DF

*p*-value: < 2.2e-16

*Regression analyses on DFW rates, all students*

We employed the same approach, including the same predictor variables, to model variation in DFW rates among terms in the study, except that we removed transfer status from the final model as preliminary analyses indicated that it did not explain a significant amount of variation in DFW rates, and used all three factors from the principal component analysis of ACT scores, instead of just the first factor, as we found that it improved AIC. We used a general linear model with a binomial distribution to create a logistic regression capable of predicting a binary success/failure outcome. The full logistic regression model was:

*DFW rate ~ ACT-Factor1 + ACT-Factor2 + ACT-Factor3 + Gender + Treatment*

A summary of the regression statistics is provided in Table C; this is the analysis plotted in Figure 1B.

**Table C. Regression statistics for DFW rates by treatment.**

This is the analysis plotted in Figure 1B.

Estimate Std. Error z value Pr(>|z|)

(Intercept) 4.58 0.46 9.89 < 0.001

ACT-Factor1 0.02 0.00 7.52 < 0.001

ACT-Factor2 0.01 0.01 1.99 0.05

ACT-Factor3 -0.01 0.01 -1.71 0.09

Gender 0.19 0.17 1.14 0.25

Experiment 1 -0.42 0.23 -1.83 0.07

Experiment 2 -0.18 0.22 -0.81 0.42

Experiment 3 -1.06 0.21 -5.15 < 0.001

Null deviance: 1118.0 on 822 degrees of freedom

Residual deviance: 945.14 on 815 degrees of freedom

AIC: 961.14

A summary of the raw failure rates for the overall student population, not controlled for variation in student characteristics, is provided in Table D. The overall failure rate during the control terms was 48%; the overall failure rate during the Experiment 3 terms was 25%.

**Table D. Raw DFW rates by term.**

Only students who took at least one exam during the semester were included in this analysis.

Treatment Year DFW rate *n*

Control Winter 2011 46.6% 191

Control Winter 2012 49.5% 182

Experiment 1 Winter 2013 40.6% 192

Experiment 2 Winter 2014 46.0% 202

Experiment 3 Winter 2016 20.4% 181

Experiment 3 Winter 2017 36.7% 98

Experiment 3 Fall 2017 19.0% 73

*Regression analyses to assess the impact of treatment on exam performance and DFW rates for URM students*

To analyze whether the experimental treatments had an impact on achievement gaps in exam performance and/or failure rates, we ran the same models as above but started with all predictors, including a term for URM status and the URM*Treatment interaction. In these models the reference values were No for URM status, meaning that positive estimates indicate that URM students had higher values than non-URM students; and again Female for Gender, meaning that positive estimates (betas) indicate that males have higher values than females; No for Transfer status, meaning that positive estimates indicate that transfer students have higher values than “native” students; and Control for Treatment, meaning that positive estimates indicate that students in the treatment have higher values than students in the Control terms. Residual plots indicated that the data met the assumptions of normality.

For failure rates, the final model was:

*DFW ~ ACT-composite + URM status +Treatment + URM*Treatment*

A summary of the regression statistics is provided in Table E.

**Table E. Regression statistics for URM status as a predictor of DFW rates by treatment.**

This is the analysis plotted in Figure 2B.

Estimate Std. Error z value Pr(>|z|)

(Intercept) 3.06 0.51 6.03 < 0.001

ACT composite 0.02 0.00 7.52 < 0.001

Experiment 1 -0.34 0.30 -1.13 0.26

Experiment 2 -0.16 0.29 -0.53 0.59

Experiment 3 -0.70 0.27 -2.62 < 0.01

URM 1.47 0.33 4.50 < 0.001

Exp1:URM -0.55 0.50 -1.10 0.27

Exp2:URM -0.18 0.50 -0.36 0.72

Exp3:URM -0.96 0.44 -2.17 0.03

Null deviance: 1071.9 on 788 degrees of freedom

Residual deviance: 877.54 on 780 degrees of freedom

AIC: 895.54

For exam scores, the best model that emerged from model selection was:

*Percentage exam points earned ~ ACT-composite + Gender + Transfer status + URM*Treatment*

A summary of the regression statistics is provided in Table F.

**Table F. Regression statistics for URM status as a predictor of exam scores by treatment.**

This is the analysis plotted in Figure 2A.

Estimate Std. Error t value Pr(>|t|)

(Intercept) 23.37 2.80 8.35 < 0.001

PC1 -0.26 0.02 -16.05 < 0.001

Gender 3.09 0.97 3.19 0.001

Trans 2.83 1.29 2.20 0.03

URM -5.02 1.82 -2.75 < 0.01

Experiment 1 -1.92 1.67 -1.15 0.25

Experiment 2 -2.45 1.67 -1.47 0.14

Experiment 3 3.07 1.40 2.20 0.03

Exp1:URM -2.37 2.82 -0.84 0.40

Exp2:URM 1.50 2.82 0.53 0.59

Exp3:URM 1.99 2.42 0.82 0.41

Residual standard error: 11.75 on 669 degrees of freedom

Multiple R-squared: 0.42; Adjusted R-squared: 0.41

F-statistic: 48.66 on 10 and 669 DF

*p*-value: < 2.2e-16

We also analyzed the data with and without controlling for ACT as an index of student preparation and ability, to assess the “raw” or “transcriptable” changes in student exam performance over time; results are shown in Table G.

**Table G. Regression statistics for URM status as a predictor of exam scores by treatment, without controlling for ACT scores.**

Estimate Std. Error t value Pr(>|t|)

(Intercept) 65.16 1.19 54.79 < 0.001

Gender 3.88 1.01 3.84 < 0.001

Trans 1.56 1.10 1.42 0.16

URM -13.67 1.87 -7.31 < 0.001

Experiment 1 -1.73 1.67 -1.04 0.30

Experiment 2 -3.03 1.71 -1.77 0.08

Experiment 3 3.93 1.44 2.73 < 0.01

Exp1:URM -1.29 3.09 -0.42 0.678

Exp2:URM 4.73 3.04 1.56 0.12

Exp3:URM 5.19 2.60 2.00 0.05

Residual standard error: 11.75 on 669 degrees of freedom

Multiple R-squared: 0.42; Adjusted R-squared: 0.41

F-statistic: 48.66 on 10 and 669 DF

*p*-value: < 2.2e-16

**References**

[1] Burnham KP, Anderson DR (2003). Model Selection and Multimodel Inference: A Practical Information-Theoretic Approach (2nd ed.). *New York: Springer-Verlag*.
