# Supplementary material for: True Grit: Passion and persistence make an innovative course design work
Source: PLoS Biol. 2019 Jul 18;17(7):e3000359. doi: 10.1371/journal.pbio.3000359 (PMC6667208; doi:10.1371/journal.pbio.3000359)
Supplement: S5 Text — (DOCX) [file pbio.3000359.s005.docx]

**S5 Text. Time-on-task survey and Student evaluations of teaching.**

All students enrolled every semester were asked to complete the standard Eastern Michigan University course evaluation form. These anonymous evaluations were completed in class during the last week of the semester in accordance with protocols established by the University. In addition, all students enrolled during the Experiment 1-3 semesters were asked to complete a 20-question survey on the average number of hours spent per week on various activities during that term (S9 Data). Two questions asked about the number of courses and course credits each student was taking, with the remaining questions asking how many hours per week, on average, the student was devoting to various activities (studying biology, studying for other courses, travel to/from campus, working, volunteering, sports, leisure time, etc.). Survey responses were only analyzed from students who were included in the analysis of student exam scores; students who reported studying biology more than 30 hours/week were removed from this data set.

Data from the three experimental treatments is provided in Table S5.

**Table S5. Time on task.**

The means reported here are of student self-reports of hours spent studying for introductory biology in a typical non-exam week.

|  | *N* (student respondents) | Mean ± S.E. |
| --- | --- | --- |
| Experiment 1 | 135 | 7.3 ± 0.38 |
| Experiment 2 | 146 | 6.9 ± 0.37 |
| Experiment 3 | 143 | 5.2 ± 0.31 |
