# Supplementary material for: True Grit: Passion and persistence make an innovative course design work
Source: PLoS Biol. 2019 Jul 18;17(7):e3000359. doi: 10.1371/journal.pbio.3000359 (PMC6667208; doi:10.1371/journal.pbio.3000359)
Supplement: S6 Text — (DOCX) [file pbio.3000359.s006.docx]

**S6 Text. Example of a typical class session during Experiment 3.**

The first author’s Introductory Biology I course meets twice a week, for 75 minute sessions. For each class session, the course website provides students with (1) a link to the pre-class video, (2) a .pdf file of the PowerPoint slides from the video, (3) a set of Potential Warm-Up Quiz questions, and (4) a .pdf file of the in-class PowerPoint slides, and (5) a copy of any in-class worksheets.

The class session on DNA Structure and Replication is described here in detail as an example. Before class, students watch the video made by the first author (file S7) and complete the accompanying potential Warm-Up quiz questions (Box 1). For this particular topic, some PowerPoint slides in the video show images from a published case study [1] and the textbook [2].

At the beginning of class, each student is given a blank half-sheet of blue paper for the quiz. Two questions from the set of potential warm-up quiz questions on this topic (Box 1) are displayed on a PowerPoint slide. Students are given three minutes to write the answers on their blue sheet. Students then exchange quiz sheets with another student in the classroom as directed. The instructor (the first author) varies the exchanges, sometimes asking students to pass their quizzes behind them, sometimes passed to the person located two chairs to the right, etc. The answers to the quiz are then displayed on a PowerPoint slide, and students grade each other. During the grading time, students are given the opportunity to raise their hand for assistance from the instructor or one of the teaching assistants. Quizzes are then passed back to their owners. The owners are given the opportunity to raise their hand to have the instructor or a teaching assistant review the grade given. Students then self-enter their quiz score, using their clicker. All of the quiz sheets are then handed in to the instructor. The entire warm-up quiz process typically takes ~12 minutes.

Announcements are made after the quiz to give dates for upcoming homework assignments and exams and re-iterate times for office hours. The class continues with the in-class PowerPoint slides (file S8), which are structured to provide many opportunities for students to answer questions and solve problems. For this particular topic, the information that students learned about DNA replication from the pre-class video is used during the in-class time to understand the process of polymerase chain reaction (PCR). During class, the instructor verbally draws out from students the similarities and differences between cellular replication and PCR. On think-pair-share activity slides, the first author uses random call for student responses for the “share” portion. On clicker question slides, students first answer alone, then are given time to talk to their neighbors about the question, and then the first author uses random call for student responses. Note, the slides that have clicker questions are not included on the in-class PowerPoint file shared with students on the course website. For this particular topic, some of the in-class work and questions are drawn from a published case study [1]. Images from the textbook [2] are also shown on some PowerPoint slides. It takes ~40 minutes to work through the slides on this topic.

The last ~20 minutes of this particular class session are spent on a worksheet, which students complete in groups (Box 2). During this time, the instructor and the teaching assistants walk through the classroom and talk with the groups to help them with the worksheet. Shortly before the end of class, the instructor reveals the correct answers for the worksheet.

**Box 1. Potential Warm-Up Quiz questions**

The questions associated with the class session on DNA Structure and Replication are shown.

| 1. Be able to draw and label, from memory, a DNA nucleotide monomer. 2. What kind of bonds join individual nucleotides to their neighbors in a single strand of DNA?   Are these strong bonds, yes or no?   1. What kind of bonds join bases to hold two strands of DNA together?   Are these strong bonds, yes or no?   1. In DNA, the bases are paired in specific combinations. What are these pairings? 2. What does it mean to say that the two strands in a DNA molecule are “antiparallel”? 3. Be able to draw, from memory, a simple diagram showing the chemical structure of DNA, showing the sugars, phosphates, and bases. Use the image on slide #5 of the video PowerPoint as a guide to the level of detail you need. Make sure you indicate the 5’ and 3’ end of each strand! 4. What does it mean to say that the process of DNA replication is “semi-conservative”? 5. List the four steps in DNA replication and briefly describe what happens in each step. 6. For each of the following proteins, describe their role in DNA replication:   Helicase, Single-strand binding protein, Primase, DNA polymerase I, DNA polymerase III, DNA ligase   1. List two key aspects of DNA polymerases. 2. What is the difference between the leading and the lagging strands during DNA replication? 3. What is an Okazaki fragment? 4. Given a drawing of a DNA molecule in the process of being copied (like the drawings on my white board in the video), be able to explain what the next step in the process should be. 5. Explain why the telomeres get shorter every time a chromosome is copied. |
| --- |

**Box 2. In-class group worksheet**

The group worksheet for the class session on DNA Structure and Replication is shown.

| **BIO 110 In-Class Group Work: DNA Replication**  *****************************************************  *NOTE: For the multiple choice questions below, more than one answer may be correct. Circle all answers that your group considers appropriate to addressing the question.*  *****************************************************  1. What would happen to DNA replication if the helicase enzyme did not function?   1. The leading strand could be copied, but not the lagging strand 2. Replication would occur, but the Okazaki fragments would not be joined together at the end 3. Replication would not occur at all 4. The RNA primers would not be removed   2. During DNA replication, the leading and lagging strands differ in that:   1. The leading strand is synthesized at twice the rate of the lagging strand 2. The leading strand is synthesized continuously, while the lagging strand is synthesized in short fragments 3. The leading strand does not require primase, while the lagging strand does require primase 4. The leading strand does not require single-strand binding protein, while the lagging strand does require this protein   3. Place in order the following steps involved in PCR.  ________ Heat to 94 degrees to separate the strands of target DNA  ________ DNA polymerase adds new nucleotide bases to copy template  ________ Repeat cycle, so that the newly synthesized strands act as templates  ________ Lower temperature to 50 degrees  ________ Raise temperature to 72 degrees  ________ Primers anneal to the template DNA  4. If one copy of a template DNA is put in a tube for PCR, then after 4 cycles of PCR, there will be ________ total copies.    5. Investigators suspect that a store is illegally mis-labeling fish for sale (swapping inexpensive fish for more desirable species). For their investigation, they used PCR to produce multiple copies of a gene in DNA harvested from the fish. Which of the following is true about the first copy of the gene produced during PCR?   1. It is composed entirely of new DNA 2. It is composed of cytosine, guanine, uracil, and adenine 3. Half is composed of original DNA, half is composed of new DNA 4. Helicase enzyme was required before the gene could be replicated by PCR   6. For their PCR reaction, the investigators mixed together: DNA from the fish, DNA polymerase, nucleotides A, T, G, and C, and one primer that is complementary to a region on the right side of the gene they wanted to amplify. However, after heating and cooling the tube 32 times, the gene was not amplified. What’s wrong?   1. They forgot to add primase 2. They forgot to add SSBP (single-strand binding protein) 3. They added the wrong template DNA 4. They forgot to add a second primer complementary to a region on the left side of the gene   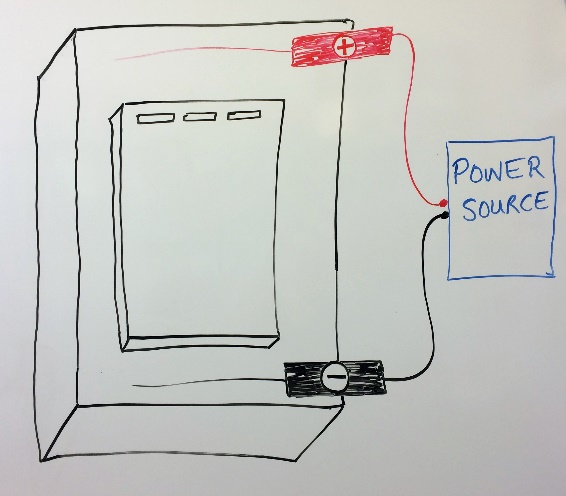  7. They loaded the DNA from their PCR in the gel as shown, and turned on the power. But when they later looked at their gel, there was no DNA in it! You correctly tell them that when they turned on the power, all their DNA all migrated out of the top of their gel. Why did this happen?  Diagram drawn  By Anne Casper |
| --- |

References

[1] Armstrong N, Platt T, Brickman P. *The Case of the Druid Dracula: Clicker Case Version.* (2009) National Center for Case Study Teaching in Science, University at Buffalo, State University of New York.

[2] Urry L, Cain M, Wasserman S, Minorsky P, Reece J. *Campbell Biology in Focus* (Second Edition) (2005). Boston: Pearson.
