# Supplementary material for: True Grit: Passion and persistence make an innovative course design work
Source: PLoS Biol. 2019 Jul 18;17(7):e3000359. doi: 10.1371/journal.pbio.3000359 (PMC6667208; doi:10.1371/journal.pbio.3000359)
Supplement: S1 PowerPoint — (PDF) [file pbio.3000359.s008.pdf]

DNA Structure and Replication

Objectives:
1. Draw how nucleotides are arranged in DNA.
2. Explain how DNA is replicated.
3. Explain the research technique of gel electrophoresis.
4. Explain the research technique of PCR.

The Case of the Druid Dracula

Image of landscape from Wales

Adapted from Armstrong N, Platt T, Brickman P. (2009)
The Case of the Druid Dracula
National Center for Case Study Teaching in Science, University at Buffalo, State University of New York.

Used with permission of NCCSTS.

The Crime

In Wales in 2001, 90-year-old Mabel Leyshorn was murdered.

Her murder had been not only brutal, but also *creepy*.

It appeared as if the Mabel's blood had been collected in a small kitchen saucepan and tasted, and a candlestick and a pair of crossed pokers had been arranged near the body.

Image of creepy candlesticks

- from BBC's *Crimewatch* December 2001

Further investigation indicated that this was no supernatural villain: The murderer had left distinctive footprints under the glass door, which had been shattered.

Moreover, the windowsill had bloodstains on it.

Of what use is the blood at the crime scene?

Image of broken glass in window

Think-Pair-Share
Review: DNA is an Organic Macromolecule

Image of train boxcars

What are the four classes of organic macromolecules?

What is the monomer of DNA?

What is the name of the general type of reaction that links monomers together?

CQ: Below is one strand from part of a DNA helix. What is the nucleotide sequence of the complementary strand?

5′-CCCTGGGCTCT-3′

A. 3′-ACTGTTAGATT-5′
B. 3′-GGGACCCGAGA-5′
C. 5′-GGGACCCGAGA-3′
D. 3′-CCCTGGGCTCT-5′
E. 5′-CCCTGGGCTCT-3′

CQ: If a sample of DNA contains 40% guanine, how much of the sample would be composed of thymine?

A. 10%
B. 20%
C. 40%
D. 60%
E. Cannot determine from the information given

Return to: The Crime Scene

Most DNA is the same from person to person. What varies in DNA that is helpful in solving crimes?

Image of drippy blood

Example: Amelogenin Gene

- Tooth enamel development
- Copies on X and Y chromosomes
- X copy is shorter than Y copy

--- indicates missing bases

Y: 5′CCCTAGGGTCTATAACGCCTAGTGTGTTGATTC 3′
3′GGGATCCCAGATATTGCGGATCACACAACTAAG 5′

X: 5′CCCTAGGGTCTA GTGTGTTGATTC 3′
3′GGGATCCCAGAT CACACAACTAAG 5′

Gel Electrophoresis

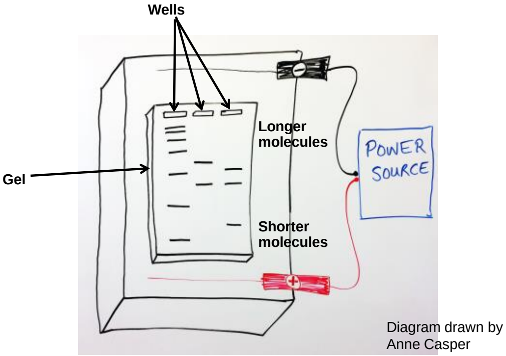

Diagram drawn by Anne Casper

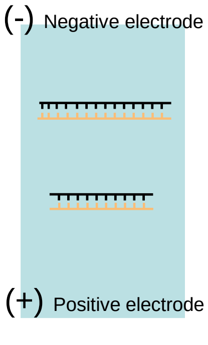

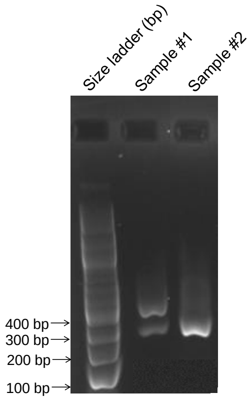

Photograph taken by Anne Casper

CQ: Is the blood at the crime scene from a female or a male?

A. female
B. male

Think-Pair-Share
Why are some bands brighter than others?

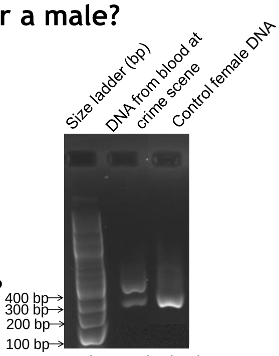

Photograph taken by Anne Casper

Return to: The Crime Scene

To see DNA on a gel, we need to have many copies of it.

How does the cell copy its DNA?

What technique can we use to make many copies of the amelogenin gene in the lab?

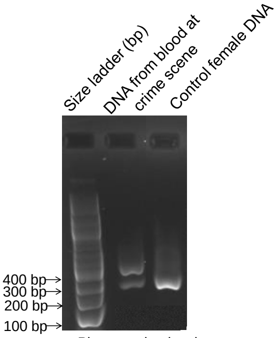

Photograph taken by Anne Casper

Leading and Lagging Strands

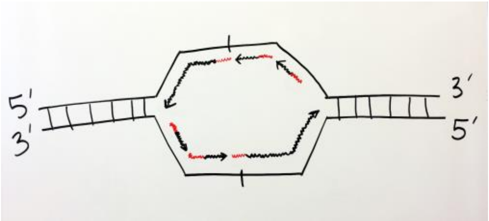

Diagram drawn by Anne Casper

Replication: The big picture

Image of a replication fork and associated proteins (Helicase, Single-strand binding protein, Primase, Pol I, Pol III, Ligase)

CQ: How would replication be affected if DNA polymerase III is not available?

A. The template strands would not be able to separate.
B. The template strands would separate but RNA primers would not be produced.
C. RNA primers would be produced but not complementary DNA.
D. There would be small breaks in the DNA backbone after replication.

CQ: Which statement is true of DNA synthesis on *both* the leading and lagging strands?

A. Synthesis progresses in a direction leading “away from” the replication fork
B. Nucleotides are added the 5’ end of an existing strand
C. Okazaki fragments are produced
D. The action of primase is required
E. A template strand is not required

PCR

Four Components

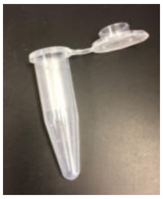

Photograph taken by Anne Casper

PCR

Photograph taken by Anne Casper

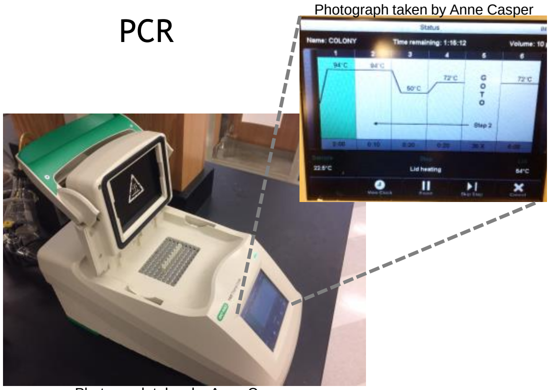

Photograph taken by Anne Casper

Videos of PCR

Cold Spring Harbor 3-D animation library

- https://www.dnalc.org/resources/3d/19-polymerase-chain-reaction.html

Just for fun – The PCR Song (by Bio-Rad)

- https://www.youtube.com/watch?v=x5yPkxCLads

PCR cycle

Step 1: Denaturation

Image of denatured DNA template

PCR cycle

Step 1: Denaturation

Image of denatured DNA template

Step 2: Annealing

Image of primers attached to template

PCR cycle

Step 1: Denaturation

Image of denatured DNA template

Step 2: Annealing

Image of primers attached to template

Step 3: Extension

Image of nucleotides added to primers

Image of two copies of template

PCR cycle 2

Image of four copies of template

PCR cycle 3

Image of eight copies of template

Each cycle doubles the number of copies

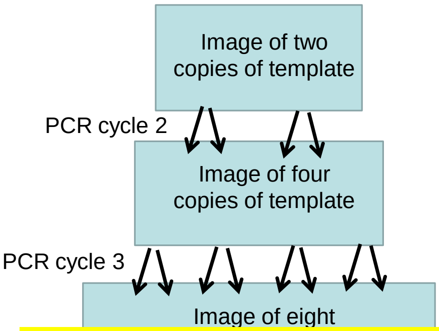

In 32 cycles at 100% efficiency, 1.07 billion copies of targeted DNA region are created

Each cycle doubles the number of copies

Think-Pair-Share

For PCR, why do researchers use the DNA polymerase from *Thermus aquaticus*, which lives in hotsprings at 70ºC, instead of *E. coli*, which lives in the human gut at 37ºC and is much-better studied?

Use PCR to amplify the Amelogenin Gene

- Tooth enamel development
- Copies on X and Y chromosome
- X copy is shorter than Y copy

```
Y:  5'CCCTAGGGTCTATAACGCCTAGTGTGTTGATTC 3'
    3'GGGATCCCAGATATTGCGGATCACACAACTAAG 5'

X:  5'CCCTAGGGTCTAGTGTGTTGATTC 3'
    3'GGGATCCCAGATCACACAACTAAG 5'
```

CQ: You need many copies of the amelogenin gene, which you will make using PCR. Which of the following do you <u>NOT</u> need to add in your PCR reaction?

A. Add DNA isolated from white blood cells
B. Add short stretches of single stranded DNA complementary to the sequence at either end of the gene.
C. Add DNA primase enzyme.
D. Add DNA polymerase enzyme.
E. Add A, T, G, and C nucleotides.

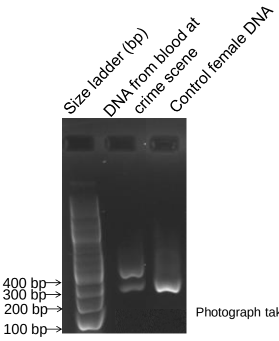

Photograph taken by Anne Casper

Is the amelogenin gene test enough evidence to convict a suspect?

Additional Markers

This will not be on the exam

Short Tandem Repeats (STRs)

- **Chromosomes 11 of person 1:**

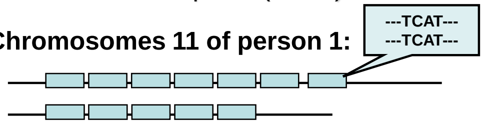

- **Chromosomes 11 of person 2:**

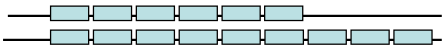

- **Different people have different numbers of repeats on their chromosomes**

The Rest of the Story…

- Standard police work identified Matthew Hardman as a suspect.
- During the arrest, a knife was found in his coat pocket. Subsequent DNA testing revealed two sources of DNA on the knife, one from Hardman and one matching the victim. The possibility of a random match was one in 73 million.
- A search of Hardman’s dwelling produced magazines and evidence of accessing internet sites featuring how to become a vampire.
- Matthew Hardman was found guilty of murder on August 2, 2002, and sentenced to life imprisonment.

In groups:
Worksheet on DNA Replication
